# Lung Involvements in Rheumatic Diseases: Update on the Epidemiology, Pathogenesis, Clinical Features, and Treatment

**DOI:** 10.1155/2018/6930297

**Published:** 2018-05-08

**Authors:** You-Jung Ha, Yun Jong Lee, Eun Ha Kang

**Affiliations:** Division of Rheumatology, Department of Internal Medicine, Seoul National University Bundang Hospital, Seongnam, Republic of Korea

## Abstract

Lung illness encountered in patients with rheumatic diseases bears clinical significance in terms of increased morbidity and mortality as well as potential challenges placed on patient care. Although our understanding of natural history of this important illness is still limited, epidemiologic knowledge has been accumulated during the past decade to provide useful information on the risk factors and prognosis of lung involvements in rheumatic diseases. Moreover, the pathogenesis particularly in the context of genetics has been greatly updated for both the underlying rheumatic disease and associated lung involvement. This review will focus on the current update on the epidemiologic and genetics features and treatment options of the lung involvements associated with four major rheumatic diseases (rheumatoid arthritis, systemic sclerosis, myositis, and systemic lupus erythematosus), with more attention to a specific form of involvement or interstitial lung disease.

## 1. Introduction

The lung is a frequent target of autoimmune mediated injury in patients with rheumatic diseases. Rheumatic disease associated lung involvement is a major determinant of morbidity and mortality in these patients. It shows a considerable heterogeneity in incidence and prevalence, severity, and the components of the involved lung structure depending on the underlying rheumatic disease and each rheumatic disease is associated with a characteristic pattern of their lung disease (Table 1) [1]. For example, up to 70~90% of patients with systemic sclerosis (SSc) or myositis exhibit lung involvement in the form of interstitial lung disease (ILD) while, in rheumatoid arthritis (RA) and systemic lupus erythematosus (SLE), the spectrum of pulmonary manifestations is quite broad involving almost every component of the lung structure or upper airway tracts. Furthermore, the clinical manifestation and severity of lung disease vary from subclinical abnormality to respiratory failure and death even within patients suffering the same rheumatic disease. The pulmonary manifestation could be the first clue to predict future or diagnose underlying rheumatic disease or it could occur later during the disease course. Although autoimmune mediated lung injury is thought to be a common mechanism, the key immune cells and cytokines driving the lung disease could be different depending on the underlying rheumatic disease.

Among many diverse forms of rheumatic disease associated lung involvements, most common is ILD. The histopathologic classification of rheumatic disease associated ILD (RD-ILD) follows 2013 revised American Thoracic Society/European Respiratory Society classification of idiopathic interstitial pneumonia, including usual interstitial pneumonia (UIP), nonspecific interstitial pneumonia (NSIP), cryptogenic organizing pneumonia (COP), diffuse alveolar hemorrhage (DAH), and others [2]. The histologic hallmark of UIP is a temporal heterogeneity with alternating areas of normal lung, interstitial inflammation, fibroblast foci, and honeycomb changes, which correspond to high resolution computed tomography (HRCT) findings of peripheral basilar predominant reticular abnormalities, honeycombing, traction bronchiectasis, and minimal to no ground glass opacities (GGOs) [3]. On the other hand, NSIP shows a relatively uniform appearance at low magnification due to a cellular interstitial infiltrate of mononuclear inflammatory cells associated with varying degrees of interstitial fibrosis. These histologic changes correspond to HRCT findings of reticulation and GGOs with little or no architectural distortion and honeycombing. In rheumatic diseases, the predominant type of ILD was found to be NISP and UIP [4–6]. It has been recognized that rheumatic disease associated UIP (RD-UIP) shows better prognosis than idiopathic UIP or idiopathic pulmonary fibrosis (IPF) [7]. Unlike idiopathic ILD where NSIP was found to show better survival than UIP, RD-NSIP and RD-UIP have shown similar prognosis [7, 8] except for RA-ILD. Thus, it would be worth looking for evidence of rheumatic diseases in patients newly diagnosed with IPF.

In this review, we will introduce the updated knowledge on epidemiology, genetics based pathogenesis, clinical characteristics, and treatment options of rheumatic disease associated lung involvement, focusing on RA, SSc, myositis, and SLE.

## 2. Lung Involvement in Rheumatoid Arthritis (RA)

The most striking feature of RA lung involvement is that almost all components of the lung structure are potential targets of injury [1]. Parenchymal diseases include ILD or nodules while airway diseases include but are not limited to bronchiectasis (BE), bronchiolitis (associated with air trapping), and bronchial wall thickening [9–11]. Pleural and vascular structures can also be affected. The prevalence of a specific form of lung disease varies depending on the population studied and the modalities used. When examined by HRCT, lung abnormalities have been found in 50–70% of unselected RA patients [9–11], among which ILD and BE are most common. However, these abnormalities are, very often, not associated with any symptoms [10, 11]. The majority of lung diseases during the course of RA occur during the first 5 years after RA diagnosis [12, 13], with the airway disease being the earliest manifestation [14]. The presence of ILD calls for particular attention since it leads to significant morbidity and mortality [15]. Airway diseases such as BE frequently occur in RA, but clinically severe cases are rare [16].

### 2.1. RA Associated ILD (RA-ILD)

#### 2.1.1. Epidemiology and Clinical Characteristics of RA-ILD

The prevalence of clinically significant RA-ILD occurs in about 10% of RA patients [17] and in up to 58% if subclinical RA-ILD is included [9, 13, 18]. RA-ILD can occur before and throughout the course after the diagnosis of RA: 34% of RA-ILD occurred within 1 year before or after RA diagnosis [19] and the risk of RA-ILD increased with RA duration [20, 21]. The lifetime risk of developing ILD has been estimated to be 7.7% in RA patients compared to 0.9% in general population [15]. Risk factors for RA-ILD identified in most studies include male gender, older age at onset, smoking, and high titer of rheumatoid factor (RF)/anti-citrullinated protein antibodies (ACPA) [22, 23] while RA duration and severity have been identified in some but not all studies [20, 21]. Unlike other rheumatic diseases where NSIP is the predominant histopathologic pattern, UIP accounts for almost half of RA-ILD patients, followed by NSIP [5, 24]. The clinical course of RA-ILD is highly variable, ranging from asymptomatic to rapidly progressive. Once clinically present, symptoms usually progress over time, albeit with different rates of progression depending on the histopathologic patterns of ILD and other clinical characteristics such as extent of disease and rate of pulmonary function decline. In general, UIP pattern, more extensive disease, and rapid decline of pulmonary function during follow-up were found to associate with poor prognosis [23, 25]. The hazard ratio (HR) of death in patients with RA-ILD was 3 times that of patients without [15]. Five-year mortality rate ranges from 35% to 39% after ILD diagnosis [15, 19].

Acute exacerbation (AE), a distinct fatal condition with rapid deterioration of respiratory status initially recognized in IPF, has been reported in ILD of rheumatic diseases including RA-ILD [26]. Radiographically, it is characterized by new development of GGOs or consolidations superimposed on the underlying reticular abnormalities. In RA-ILD, this condition was associated with 2.5-fold increase of mortality showing 64% of them dying during initial exacerbation [27]. UIP, old age at ILD diagnosis, and methotrexate usage were associated with AE of RA-ILD.

UIP pattern in HRCT was found to show different clinical course from non-UIP RA-ILD [28]. The former was associated with more frequent hospitalization and oxygen therapy and rapid decline of pulmonary function [29]. When the median survival time for all RA-ILD subjects was 5 years, RA-UIP had a worse median survival time than RA-non-UIP (3.2 versus 6.6 years), which did not differ from that of IPF [28]. The high prevalence of UIP partly explains high mortality rate in patients with RA-ILD.

#### 2.1.2. Genetics of RA and RA-ILD

Until now, the strongest genetic factor associated with RA has been shared epitope (SE) or certain HLA-DRB1 alleles that share a conserved amino acid sequence at positions 70–74 of the beta chain. These alleles were found to have higher binding affinity to citrullinated proteins than nascent proteins [30], which partly explains the underlying mechanism of association between RA specific antibodies or ACPAs and SE [31]. Smoking induces protein citrullination in the lung tissue [32]. The interaction between smoking and SE has been found to ultimately attribute to ACPA positive RA development [33]. This gene-environmental interaction is further supported by the gene-dose effect observed with anti-CCP positive RA development [33].

**Figure 1:**
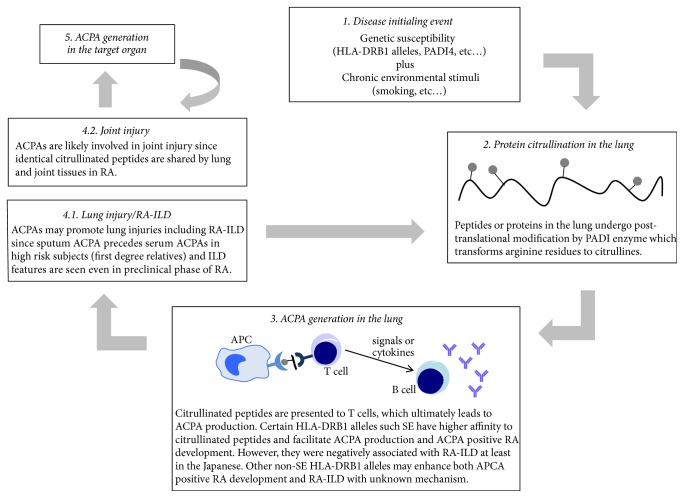
Schematic presentation of shared pathogenesis of RA and RA-ILD.

The strongest predictors of RA-ILD are RF and/or ACPA positivity and their higher titers correlate with more severe ILD [22]. Smoking has been a well-recognized risk factor not only for RA but also for RA-ILD [22]. RA and RA-ILD share common disease predictors (RF and ACPA) and an environmental risk factor (smoking) particularly affecting lung tissue. These findings may suggest that RA begins in the lung at least in smokers and that RA associated lung diseases including RA-ILD are the consequence of the same gene-environmental interaction as in RA: smoking may be a triggering insult to induce pulmonary APCA production that subsequently contributes to RA and RA-ILD development (Figure 1). Further findings that reinforce this hypothesis are as follows: (1) ACPA is locally produced in the lung tissue in early RA [34], (2) the pulmonary positivity of ACPAs or RF precedes serum positivity in high risk subjects [35], (3) ACPAs are associated with both ILD and airway disease in preclinical RA phase [36], (4) identical citrullinated peptides are shared in bronchial and synovial tissues in RA patients [37], and (5) a broader epitope is spreading in patients with RA-ILD than RA alone [38]. More compelling evidence to support this hypothesis has come from a study by Restrepo et al. where the association between RA-ILD and smoking was restricted in SE carriers [20].

However, the role of SE in RA-ILD pathogenesis seems to be more intriguing. Although SE has been shown to interact with an environmental risk factor to produce ACPA and the presence of ACPA is a strong predictor of RA-ILD, the association between SE and RA-ILD is not as straightforward as the association between SE and ACPA positive RA. SE was found to associate with deceased risk of RA-ILD in the Japanese [39]. Instead, HLA-DR2 serological group (HLA-DRB1^*∗*^15 and ^*∗*^16) was associated with increased risk of RA-ILD [39, 40] and RA-UIP [41] in the Japanese. On the other hand, the distribution of SE was similar in RA versus RA-ILD in a UK population and there was an increased risk for RA-ILD by HLA-DRB1^*∗*^07 [42]. The HLA-DRB1 alleles associated with RA-ILD might not be exactly SE alleles but they might also contribute to ACPA production. Likewise, each SE allele may differ in their roles in RA-ILD development although their roles in ACPA positive RA development are quite in the same direction. Recently, Song et al. explored polymorphisms in PADI4 and HLA-DRB1 in RA patients and found that recessive genotype of padi4_92 was associated with airway abnormalities and that tryptophan at position 9 of HLA-DRB1 amino acid sequence was strongly associated with RA-ILD in Korean patients with an odds ratio (OR) of 22.89 [43].

Although many RA susceptibility genes have been identified by genome wide association study (GWAS), few have been validated in RA-ILD subsets. A whole exome sequencing study has found that mutations in familial pulmonary fibrosis-linked genes (TERT, RTEL1, PARN, or SFTPC) are shared with RA-ILD [44].

#### 2.1.3. Treatment of RA-ILD

The treatment for RA-ILD is quite empirical, because there have been no randomized placebo-controlled trials. Physicians generally follow the treatment strategies used for the corresponding type of idiopathic ILD. When encountered with AE of RA-ILD, moderate-to-high dose steroid therapy (1 mg/kg oral prednisone or its equivalent) with or without another immunosuppressive agent is usually administered. Commonly used immunosuppressants in combination with steroids are azathioprine, cyclophosphamide (CYC), or mycophenolate mofetil (MMF). A recent study of 125 patients with RD-ILD receiving MMF, including 18 with RA-ILD, showed modest improvement in forced vital capacity (FVC) and diffusing capacity of carbon monoxide (DLCO) and reductions in prednisone dosage [45]. Rituximab treatment was successful for severe refractory RA-ILD in a small case series [46], whereas a recent single-center experience with rituximab for RD-ILD described inappreciable effect [47]. Perfenidone, an antifibrotic drug of unknown mechanism approved for IPF, might benefit RA-UIP phenotype, and studies are currently ongoing. Patients with non-UIP histopathologic patterns are more likely to respond to steroid/immunosuppressants. All patients with RA-ILD should be encouraged to cease smoking.

### 2.2. RA Associated Airway Diseases

Airway involvement is prevalent in 39–60% of patients with RA when assessed by HRCT [10, 13]. Both upper and lower airways can be involved. The most common form of upper airway involvement is cricoarytenoid arthritis, found in nine out of fifteen RA patients by neck HRCT but only two by laryngoscopy, suggesting clinically significant involvement is infrequent [48]. Symptoms are variable including hoarseness, odynophagia, or dysphonia, but rarely prominent in most of the cases. However, it can cause upper airway obstruction, requiring immediate endotracheal intubation.

Among RA associated lower airway diseases, BE and bronchiolitis have long been recognized to associate with RA [49, 50]. BE refers to the permanent irreversible dilatation of cartilage-containing airways characterized by recurrent cough, sputum production, and respiratory infections. Abnormally dilated bronchi lead to impairment of host defenses, chronic bacterial infection, and airways inflammation, forming a vicious cycle [51]. A significantly higher prevalence (2.7%) of symptomatic BE in RA patients has been noted compared to 0.03% in the general population [16]. HRCT detects BE at much higher frequencies in up to 30% of patients [10]. The temporal relationship between BE and RA has been a debate for a long time. BE far preceding RA has been reported in many studies while, in others, BE has been recognized as a late complication of RA [52, 53]. These conflicting reports are probably because most of the old studies rely on symptom onset of respiratory and articular systems. Recent HRCT studies have demonstrated a high prevalence of subclinical airway diseases resembling BE at the first diagnosis of RA or in preclinical RA [14, 54]. Similar to ILD, high disease activity and autoantibody positivity were found to associate with BE in RA [55]. The coexistence of RA and BE has significant health implications in terms of infectious complications especially under treatment with disease modifying antirheumatic drugs (DMARDs). RA patients with BE have been shown to have mortality rates 7.3 times that of the general population, five times that of patients with RA alone, and 2.4 times that of patients with BE alone [56].

The mechanism for coexistence of RA and BE is unclear. A plausible hypothesis is that chronic bacterial infection as a result of BE provides a prolonged source of antigenic stimulation that leads to breakdown of immune tolerance and subsequent development of RA in genetically predisposed individuals [57]. The association of airway disease with RA seems to be different from that of RA-ILD. As in ILD, airway disease was found in preclinical RA [36]. However, RA patients with BE tended to be nonsmokers [58] and a high prevalence of BE (25%) was identified in life-long nonsmoking RA patients [59]. Interestingly, SE was found to associate with airway disease in RA patients [41, 60]. The shared genetic risk factors in terms of SE might contribute to the association between BE and RA. On the other hand, the RA-ILD predisposing allele HLA-DRB1^*∗*^15:02 was found protective against airway disease in Japanese RA [60]. HLA-DQB1^*∗*^03:01 and HLA-DQB1^*∗*^06:01 were found as susceptibility alleles for BE in RA [41, 61]. A polymorphism in PADI4 (recessive genotype of padi4_92) was reported to associate with the airway abnormalities in RA [43]. A small study showed that 16% of RA patients with diffuse BE were heterozygous for the delta F508 mutation of the cystic fibrosis transmembrane conductance regulator gene (CFTR) regardless of sweat chloride concentration or nasal potential difference measurements [62].

Follicular bronchiolitis and obliterative bronchiolitis (also referred to as constrictive bronchiolitis) are other lower airway involvements of RA than BE. Follicular bronchiolitis is pathologically defined as lymphoid hyperplasia with reactive germ cell centers within bronchiole walls [63]. Obliterative bronchiolitis is a fibrotic condition characterized by concentric narrowing of membranous and respiratory bronchioles caused by peribronchiolar inflammation and fibrosis without evidence of lymphoid hyperplasia. The prognosis of these conditions is reported to be poor [64].

### 2.3. RA Associated Pleural Involvement

Pleural abnormalities associated with RA include pleural effusion, pleuritis, pleural nodule, pneumothorax, and fibrothorax, among which pleuritis and pleural effusions are most common. Pleural disease used to represent the most common form of lung involvement in RA but has become far less prevalent in the last two decades probably due to early diagnosis of RA and more aggressive treatment. Although autopsy studies identified pleural effusion in up to 70% [65], most of them are scanty and clinically silent. Pleural effusions in RA are exudative and sterile, often with mixed cell count (monocytes-predominant), high lactate dehydrogenase, low glucose, and low pH [65]. Most patients experience recovery along with DMARDs therapy.

### 2.4. RA Associated Vascular Disease

The representative vascular involvement in RA lung is rheumatoid vasculitis, characterized by destructive inflammatory infiltrate within small- and medium-sized blood vessel walls on pathology. However, primary vasculitic involvement of the lung is rare whereas peripheral neuropathy and cutaneous vasculitis are common. Pulmonary arterial hypertension (PAH) is extremely rare in RA.

## 3. Lung Involvement in Systemic Sclerosis (SSc)

SSc is a systemic autoimmune disease characterized by immune activation, vasculopathy, and fibrosis. Tissue fibrosis of the skin and internal organs is the hallmark of the disease that dictates the clinical course of SSc. Due to fibrosis and vasculopathy, the lung manifestation involves ILD and/or PAH, both of which are the leading causes of death, accounting for 33% and 28% of SSc associated mortality, respectively [66]. ILD and PAH can occur in both diffuse and limited subsets but in general, the former develops more frequently in the diffuse subset with anti-topoisomerase I antibody (ATA) and the latter in the limited subset with anti-centromere antibody (ACA). Other SSc-specific antibodies, anti-U3 RNP and anti-Th/To antibodies, have shown heterogeneous associations with SSc-ILD [67]. Anti-U3 RNP antibodies were found to associate with ILD and PAH in a US study and with PAH in a UK study. Anti-Th/To antibodies were found to associate with SSc-ILD and PAH [67].

### 3.1. SSc Associated ILD (SSc-ILD)

#### 3.1.1. Epidemiologic and Clinical Characteristics of SSc-ILD

It has been found that ILD is seen in up to 90% of patients with SSc depending on modalities used and patient populations studied [68]. HRCT has been considered as the gold standard detection method particularly compared to pulmonary function test, with the latter resulting in high false negative rate in early SSc-ILD: when FVC < 80% was used as a stand-alone method, 63% of SSc-ILD cases were undetected [69]. According to the analysis of the European Scleroderma Trials and Research group (EUSTAR) on 3656 patients with SSc, ILD was found in 53% in a diffuse and 35% in a limited subset by plain chest radiography [70]. Approximately 40% of patients with SSc were shown to experience moderate (FVC of 50–75%) to severe (FVC < 50%) restrictive lung disease [71], with the latter being critically associated with mortality rate of 42% within 10 years after the onset of first non-Raynaud symptom.

The clinical course in terms of lung function change has been considered highly variable in SSc-ILD [72]: when 226 SSc patients with a median disease duration of 1.6 years were followed up for median 57 months, patients (51%) with initial FVC ≥ 80% rarely showed decline, while the others (49%) with initial FVC < 80% were stable (20%), deteriorated at various rates (16%), or even improved (14%) during 72 months of estimable trajectory time. Although highly variable, there are several consistent patterns for the clinical course of SSc-ILD. Most of the lung function decline occurs during the first 3-4 years after the onset of non-Raynaud's symptom, after which the decline is rather indolent [71, 73]. The progression has been found to be best predicted by the low baseline FVC or higher extent of radiographic lung fibrosis [71–76]. Patients with normal HRCT or pulmonary functions at the time of diagnosis rarely develop ILD in the next 5 years. Other predictors of progression in some but not all studies were African-American ethnicity, cardiac involvement, male gender, early disease, abnormal nailfold capillaroscopy pattern, ATA positivity, and smoking [71–74, 77–79].

On the other hand, it has been consistently reported in a number of cross-sectional studies that esophageal dilation in HRCT is associated with more severe pulmonary function impairments [80–82]. The degree of esophageal diameter was found to negatively correlate with FVC and DLCO values [80]. Moreover, longitudinal correlations have been reported between the presence of high degree of gastroesophageal reflux (GER) and more rapid decline of pulmonary function values [81, 82]. Notably, centrilobular fibrosis and bronchocentric distribution of lung involvement were found to be very common in SSc-ILD (84% of all NSIP) and they were almost invariably associated with esophageal abnormalities [83]. This finding strongly suggests that microaspiration is one of the potential culprits to cause SSc-ILD progression. The issue whether GER has a deleterious effect on SSc-ILD progression is of high importance since effective anti-acid and/or anti-reflux therapy is available. However, both GER and SSc-ILD occur as early complications of SSc, which makes it difficult to assess their temporality for causal relationship. When examined in patients with very early diagnosis of SSc (VEDOSS), abnormal findings of esophageal involvement were prevalent with 75% of them showing abnormal pressure and speed of esophageal peristalsis [84]. Moreover, the association between esophageal involvement and positive lung HRCT or low DLCO values was observed as early as in patients with VEDOSS. GER and microaspiration have been proposed as pathogenic in IPF [85]. However, the causal mechanism can work in both directions with one in which IPF develops due to reflux of gastric contents and with the other in which GER is a consequence of distortion of mediastinal structure and greater transdiaphragmatic pressure gradient caused by IPF. Regardless of the true first trigger, a vicious cycle is created leading to accelerated lung injury, which also applies to SSc-ILD in a similar manner. To date, no prospective randomized trial has been done to address if GER treatment improves clinical outcomes of IPF. Two post hoc analyses on the placebo groups of patients with IPF from clinical trials reported contrasting results on proton pump inhibitors [86, 87]. Until now, it is not clear whether antacid treatment helps lung function preservation in IPF or ILD.

Histologic pattern of ILD was not associated with any clinical outcome [4, 7], reflecting that the extent of lung involvement and degree of pulmonary function impairment are more relevant [72–76]. The predominant pattern of SSc-ILD is NSIP based on both biopsy and HRCT [4, 68]. Although GGOs are often seen, the reversal is rarely observed during treatment, in less than 5% of the cases [88]. This finding suggests that the GGOs observed in SSc-ILD do, in fact, represent fine fibrosis compared to inflammation due to limited resolution of HRCT and are consistent with the result of the previous study where fibrotic rather than cellular NSIP constitutes the majority of SSc-NSIP (47/62) [4]. This concept is further supported by the finding that GGOs are replaced by fibrosis (honeycombing, bronchiectasis, and reticulation) regardless of therapy [89]. UIP patterns contribute to 15% of SSc-ILD but with more germinal centers and less fibroblastic foci [90], partly explaining that rheumatic disease associated UIP shows better survival than IPF.

The mortality of SSc patient, when evaluated by a meta-analysis [91], showed an overall threefold increase of standardized mortality rate: the 5-year and 10-year survival of SSc patients from diagnosis were reported to be 74.9% and 62.5%, respectively. The mortality risk among SSc patients with ILD was 2.89 times compared to those without.

#### 3.1.2. Genetics of SSc and SSc-ILD

Since the first large scale GWAS performed on SSc patients in 2010 [92], many SSc susceptibility genes within and outside of the major histocompatibility complex (MHC) region have been identified by subsequent GWAS, immunochips, and their follow-up studies [93]. MHC class II region has been the most significant susceptibility locus while non-MHC genes continue to be identified. One of the prominent features of SSc susceptibility genes outside MHC is that vast majority of them are in fact susceptibility genes of other autoimmune diseases such as SLE, RA, multiple sclerosis, inflammatory bowel disease, and primary biliary cirrhosis (PBC) [93]. When autoimmune diseases are clustered based on the number of shared susceptibility genes at GWAS level, SSc was found to most closely correlate with SLE and PBC [93].

The SSc susceptibility genes can be classified according to their roles: those involved in immune functions and inflammation and those involved in extracellular matrix (ECM) deposition and fibrosis. The functions of the former genes include autophagy/apoptosis and DNA clearance (ATG5, PPARG, FAS, RHOB, and DNASE1L3), type I interferon (IFN) signaling (IRF5, IRF7, and IRF8), IL12 signaling (IL12A, IL12RB2, IL12RB1, and STAT4), other cytokines and signaling (TNIP1, TNFAIP3, IRAK1, and TNFSF4), and adaptive immunity of B (BANK1, BLK) and T cells (CD247, CSK, and PTPN22) [94]. Although the hallmark of SSc is fibrosis, the number of GWAS genes (e.g., PPARG) directly involved in fibrosis is far less than those in immune system and inflammation. This finding partly reflects study design driven bias associated with the use of immunochips. However, the paucity of fibrosis genes found by GWAS or GWAS follow-up studies raises several scenarios: tissue fibrosis in SSc might be (1) the downstream result of immune activation and inflammation, (2) effect size failing to reach statistical significance at GWAS level, (3) associated with rare genetic variants, or (4) the result of epigenetics. Since treatment effect with immunosuppressants has been limited against skin or lung fibrosis mainly stabilizing rather than reversing it [95, 96], the first explanation looks skeptical. Candidate gene analyses, which might have advantage to capture genes with small effect size, have revealed fibrosis related genes, caveolin-1 gene and CTGF, as SSc susceptibility genes [97, 98]. A recent approach using whole exome sequencing seems to partly complement GWAS-based genetics by detecting rare coding variants involved in fibrosis. Using this method, novel SSc susceptibility genes enriched in ECM related pathway* (COL4A3, COL4A4, COL5A2, COL13A1, and COL22A1)* and SSc-ILD susceptibility gene (XRCC4) involved in DNA repair have been further identified [99]. Since the concordance rate between monozygotic twins is only 4% in SSc, one can easily expect that, in addition to genetic factors, epigenetic alterations specific to genes, cells, and tissues play an important role in SSc. Epigenetic mechanisms include DNA methylation, histone modification, and noncoding RNAs including miRNAs. Evidence is rapidly accumulating that these mechanisms are distinctively used among immune cells and tissue fibroblasts of SSc patients [100, 101]. Epigenetics in SSc will not be discussed here.

Among the SSc susceptibility genes identified by GWAS and candidate gene analyses, only a limited number of genes were investigated about their association with SSc-ILD. The IFN regulatory factor 5 gene (IRF5) encodes one of the IFN regulatory factors critical for type I IFN regulation and virus-induced immune activation. Recently, IRF5 rs2004640 T allele (also known to be associated with SLE), which creates a donor splice site in intron 1 of IRF5 leading to transcription of the alternative exon 1B, was found to be associated with SSc and SSc-ILD in a European French population [102]. IRF5 rs4728142 A allele was found to be associated with lower IRF5 expression, higher FVC at enrollment, and better survival in Caucasian SSc patients [103]. STAT4 rs757486 T allele and IRF5 rs2004640 T allele were shown to have an additive effect towards susceptibility to SSc-ILD [104]. ALOX5AP rs10507391 A allele was also found to have an association with SSc-ILD in a European population enrolled in an EUSTAR group [105]. Other genes whose polymorphisms were shown to associate with SSc-ILD include CTGF [97, 98], NLRP1 (also having an additive risk with IFR5 and STAT4 on SSc-ILD) [106], CD226 [107], and HGF [108].

Ironically, one of the most striking features of SSc-ILD genetics has come from the IPF gene studies. None of the non-MHC susceptibility genes found by IPF GWAS were associated with SSc-ILD, which contrasts the distinctive pathogenesis of SSc-ILD and IPF [109–112].

#### 3.1.3. Treatment of SSc-ILD

Since those with initial FVC ≥ 80% rarely show decline in lung function [72], treatments should be focused on symptomatic patients with moderate to severe extent or with progression. As mentioned in the previous section, GGOs found in HRCT of SSc-ILD patients may represent fine fibrosis rather than inflammation [88, 89]. In line with this finding, the main effect of anti-inflammatory or immunosuppressive treatment against SSc-ILD has been stabilizing lung function rather than improving it. In the Scleroderma Lung Study (SLS) I, 158 SSc patients who had symptomatic ILD with an evidence of active alveolitis and a FVC between 45 and 85% were randomly allocated to oral CYC versus placebo for one year and were followed for another year [95]. The mean absolute FVC difference of 2.53% at 12 months was significant in favor of CYC. The effect was maintained at 1 year off treatment with the mean absolute FVC difference of 1.95% but disappeared by 2 years off treatment [113]. In SLS II where MMF for 2 years was compared with oral CYC for one year, the two drugs showed similar results at 2 years with better safety profile for MMF [96]. Their mean FVC improved from baseline by 2.17% in MMF group and 2.86% in CYC group. However, 2017 updated EULAR recommendation still suggests cyclophosphamide preferentially over MMF based on two previous high-quality randomized controlled trials consistently showing an efficacy of CYC against SSc-ILD compared to placebo [95, 114]. MMF could be considered as a first-line treatment for patients with comorbidities and little tolerability to toxicities. In addition to traditional immunosuppressives, both tocilizumab (IL-6 receptor antagonist) and rituximab (B cell depleting agent) have shown efficacy in SSc-ILD and further investigations are underway [115, 116]. One of the tyrosine kinase inhibitors, nintedanib, affects receptor signaling of VEGF, PDGF, and FGF. It has shown efficacy against IPF and is under phase III trial for SSc-ILD. Combination treatment with pirfenidone and MMF is now being investigated for SSc-ILD.

### 3.2. SSc Associated Pulmonary Arterial Hypertension (SSc-PAH)

The pathogenic mechanisms underlying PAH are abnormal proliferation, vasoconstriction, and thrombosis of pulmonary vasculature. The presence of PAH is defined at right heart catheterization (RHC) by a mean pulmonary arterial pressure of ≥25 mm Hg with a pulmonary capillary wedge pressure of ≤15 mm Hg. However, all forms of pulmonary hypertension can occur in SSc: isolated PAH, pulmonary hypertension from left heart dysfunction, and pulmonary hypertension secondary to ILD or hypoxia. Moreover, combinations of these forms can occur in SSc. However, this review will focus on isolated PAH.

#### 3.2.1. Epidemiologic and Clinical Characteristics of SSc-PAH

PAH is a serious complication of SSc with high mortality if not promptly diagnosed and properly treated. The prevalence of SSc-PAH varies depending on the method used and populations studied. When assessed by RHC in high risk patients identified by echocardiography, DLCO patterns, or unexplained dyspnea, PAH affects 8–12% of SSc patients [117, 118]. SSc-PAH is more common in a limited than diffuse subset disease. In particular, SSc-PAH has been observed in up to 50% of CREST syndrome [119]. Unlike SSc-ILD which is an early complication of SSc in most cases, SSc-PAH occurs within 5 years from the first non-Raynaud phenomenon symptom in half of the cases and the mean interval between SSc diagnoses and PAH occurrence was 6.3 years [120].

**Figure 2:**
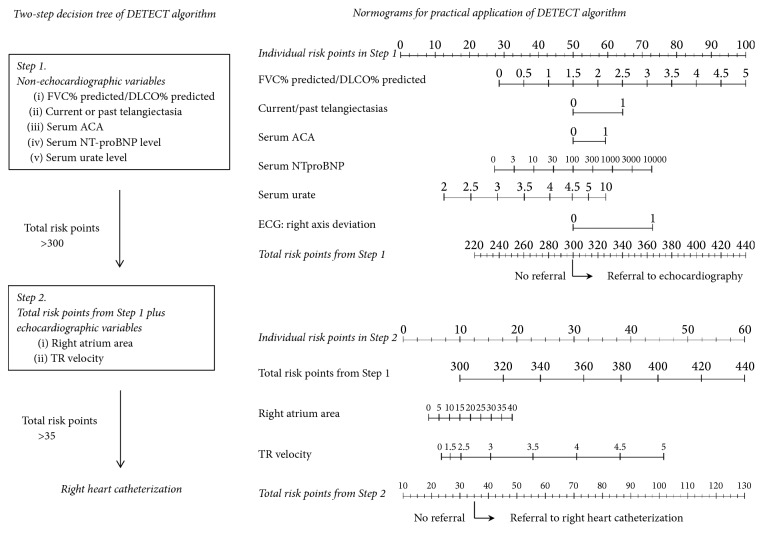
DETECT algorithm. ACA: anti-centromere antibody; DLCO: diffusing capacity of carbon monoxide; FVC: forced vital capacity; NT-proBNP: N-terminal probrain natriuretic peptide; TR: tricuspid regurgitation (cited and modified from “Evidence-Based Detection of Pulmonary Arterial Hypertension in Systemic Sclerosis: The DETECT Study” by Coghlan JG, et al. Ann Rheum Dis 2014; 73: 1340-9).

As reflected in a study where active surveillance and early treatment of PAH improve survival compared to passive identification during routine practice [121], the poor prognosis of SSc-PAH is partly attributed to delayed diagnosis due to clinically silent nature of the disease until advanced. SSc-specific risk factors for PAH include male gender, old age, presence of ACA or anti-U3 RNP, presence of telangiectasia, digital ulcers, and limited subset/CREST syndrome [122]. However, none of these risk factors are sufficient indicators of PAH. The laboratory findings highly suggesting PAH include elevated levels of N-terminal probrain natriuretic peptide (NT-proBNP) or disproportionate decrease of DLCO [123–125]. Since RHC is invasive, echocardiographic measure of tricuspid regurgitation (TR) velocity is often used as a screening tool to select patient candidates for RHC and TR velocity > 2.5 m/s has been considered as a threshold to suspect PAH. However, 20% of high risk patients with mild SSc-PAH were not detected at this threshold [126] and the sensitivity of echocardiography has ranged from 50% to 90% [127, 128]. The DETECT study proposed an algorithm to select patients under high suspicion of PAH for referral to RHC and to minimize missed or delayed diagnoses [126] (Figure 2). When compared with European Society of Cardiology/European Respiratory Society 2009 guidelines, the DETECT algorithm recommended more patients for RHC and detected more patients with PAH [129]. In DETECT study where 57 patients with SSc-PAH were followed [130], 44% (25/57) of them showed progression during a median follow-up of 12.6 months. Thirteen of the twenty-five showed mild PAH in WHO functional class I or II. Factors associated with progression were male gender, lower DLCO, higher FVC/DLCO ratio, and poor functional capacity [130].

The adjusted survival was significantly worse in ILD associated PAH than in SSc-PAH showing a 5-fold increase of mortality risk: 3-year survival rates were 39% in ILD-PAH versus 64% in SSc-PAH [131]. The overall survival in SSc-PAH has been reported as 81%, 64%, and 52% at 1 year, 2 years, and 3 years, respectively, in a meta-analysis [132]. Compared to SSc patients without PAH, patients with SSc-PAH showed more than threefold increase of mortality risk [133]. The prognosis of SSc-PAH has been found to be worst compared to idiopathic or PAH of other rheumatic diseases [134].

#### 3.2.2. Genetics of SSc-PAH

Genes identified for the risk of SSc as well as those implicated in idiopathic PAH have been pursued by large scale case-control studies using a candidate gene approach. MIF rs755622 C allele in the promoter region was associated with SSc-PAH particularly in the diffuse subset phenotype combined from multiple cohorts of European origin [135]. The SSc susceptibility gene PPARG rs10865710 C allele showed an association with SSc-PAH in a French population [136]. KCNA 5, another SSc susceptibility gene, is implicated in vascular tone regulation that its inhibition during hypoxia produces pulmonary vasoconstriction. Its variants showed conflicting result with an association with SSc-PAH in a French population [137] but failed to be replicated in other European ancestries [138]. A rare nonsynonymous TLR2 variant (Pro63His) was found to be associated with ATA positivity, diffuse subset, and increased risk of SSc-PAH development (HR = 5.61) in a European population of multiple origins [139]. This variant was found to increase the levels of IL-6 and TNF-*α*. UPAR encodes a pleiotropic receptor involved in fibrosis and vascular remodeling and its rs344781 G allele was associated with limited subset, digital ulcers, and SSc-PAH in a combined cohort of Italian and French origins [140]. G allele of rs5029939 at TNFAIP3 encoding ubiquitin-modifying enzyme has been shown to associate with diffuse subset (OR = 2.71), SSc-ILD (OR = 2.26), and SSc-PAH (OR = 3.11) in a European population of multiple origins [141]. Two SNPs at IL23R, rs11209026 G allele and rs11465804 T allele, showed positive associations with diffuse subset and ATA positivity and negative association with SSc-PAH in a US population [142]. The CXC chemokine stromal cell-derived factor 1 (SDF-1/CXCL12) and its receptor CXCR4 are involved in regulation of angiogenesis. SDF1-3′ A allele was associated with SSc-PAH (OR = 2.37) and digital ulcers (OR = 2.33) [143].

#### 3.2.3. Treatment of SSc-PAH

Active surveillance and early treatment may improve survival in SSc-PAH [121]. According to 2013 American College of Rheumatology recommendations for screening and monitoring of PAH in rheumatic diseases [144], all SSc patients, patients having SSc spectrum disorders (having sclerodactyly, nailfold capillary abnormalities, or SSc-specific autoantibodies), or those with PAH signs/symptoms (dyspnea on rest/exercise, fatigue, presyncope/syncope, chest pain, palpitations, dizziness, and lightheadedness) should undergo screening evaluations for PAH using PFTs with DLCO, echocardiography, and NT-proBNP. The panel also endorsed DETECT algorithm in SSc patients [126] if DLCO < 60% and SSc duration > 3 years from the first non-Raynaud's symptom. The recommendation criteria for RHC in these patients are shown in Table 2 [144]. In SSc and SSc spectrum disorders, annual follow-up of echocardiography and PFTs was recommended. The full screening panel (echocardiography, PFT, and NT-proBNP) should be performed as soon as any new signs or symptoms are present. The diagnosis of PAH should reply on RHC.

Vasodilating calcium channel blockers are used as the first-line treatment for SSc-ILD, but for most of the progressive cases, they are insufficient to resolve symptoms and PAH related hemodynamics. Systemic prostacyclin analogues have been used and more recently, endothelin-1 receptor antagonists and phosphodiesterase type 5 inhibitors have been used to treat PAH [145]. However, these treatments do not show survival benefits to date beyond intermediary outcomes such as symptom improvement and exercise tolerance. Among PAH of WHO group 1, SSc-PAH is generally the least responsive to therapy and has a significant mortality [134, 145].

Nevertheless, the results of several recent randomized controlled studies are promising and worth to mention. The SERAPHIN trial compared the efficacy of the long-term treatment with nonselective endothelin receptor antagonist, macitentan versus placebo in symptomatic PAH including RD-PAH. After 3 years, macitentan reduced the risk of composite end point for death due to PAH and worsening of PAH in a dose dependent manner by 30% with 3 mg treatment and 50% with 10 mg treatment [146]. The effect was consistent for RD-PAH in subgroup analysis and was significant even in those receiving background treatment with phosphodiesterase type 5 inhibitors. The AMBITION trial, comparing ambrisentan (endothelin receptor A selective antagonist) plus tadalafil (phosphodiesterase type 5 inhibitor) versus either ambrisentan or tadalafil monotherapy in treatment naïve symptomatic PAH, showed that the combination therapy reduced the risk of composite outcome of death of any cause and worsening of PAH by approximately 50% [147]. The post hoc analysis showed that this effect was consistent in both subgroups of RD-PAH and SSc-PAH [148]. In the phase 2 GRIPHON trial, selexipag (oral selective IP prostacyclin receptor agonist structurally distinct from prostacyclin) reduced the risk of composite endpoint of death or a complication related to PAH compared to placebo, with similar efficacy regardless of baseline PAH therapy [149]. Post hoc analysis on RD-PAH patients (half from SSc-PAH) from the GRIPHON trial showed 41% risk reduction for the same composite endpoint in RD-PAH and SSc-PAH [150]. These clinical trials not only show the efficacy of updated vasodilatory drugs but also imply that combination of drugs with different vasodilating mechanisms is also effective. Going one step forward, combination therapy targeting all three components of disease pathogenesis (vasculopathy, fibrosis, and autoimmunity) may be tried to improve prognosis.

## 4. Lung Involvement in Myositis

In the majority of cases, myositis-associated lung involvement takes a form of ILD. Unlike SSc, PAH is often secondary to ILD and isolated PAH is rarely found [151]. Although not a primary lung involvement of myositis, aspiration pneumonia and hypoventilatory respiratory failure are two serious complications related to pharyngeal and respiratory muscle involvement, respectively. The review will focus on myositis-associated ILD.

### 4.1. Epidemiologic and Clinical Characteristics of Myositis-Associated ILD

The prevalence of ILD ranges from 23.1 to 65% in patients with myositis depending on the modalities used [152, 153]. However, when associated with anti-aminoacyl tRNA synthetase (ARS) antibody syndrome, the prevalence exceeds 70% [154]. As in ILD of other rheumatic diseases, ILD can appear with, before, or after the onset of skin or muscle manifestations but tends to be a component of early myositis [152, 155]. Although the clinical course of myositis-associated ILD is variable, it is distinguished from SSc-ILD in that the former can be categorized in general into three clinical patterns based on respiratory symptoms at presentation [152, 156]: rapidly progressive form with acute onset symptoms, chronic form with slowly progressive symptoms, and asymptomatic or subclinical form. This is in contrast to SSc-ILD where fibrosis is the hallmark of the disease and progression occurs in a more chronic way. This difference reflects that inflammatory change is more prominent in myositis-associated ILD, generally in proportion to the rate of symptom deterioration, being most severe in rapidly progressive form where respiratory failure occurs within weeks [156].

Chronic form presenting with insidious onset dyspnea and a nonproductive cough is the most common variant (50%) and rarely shows constitutional symptoms. Up to 30% of polymyositis (PM) and dermatomyositis (DM) patients seem to have subclinical or asymptomatic ILD [152, 157]. This lack of overt symptoms emphasizes the need for pulmonary screening in all myositis patients, especially those with anti-Jo-1 antibody (=anti-histidyl tRNA synthetase antibody). However, ILD that initially presents as the aforementioned two patterns can transform into the rapidly progressive pattern during the later course of the disease. The rapidly progressive forms often take a histopathologic finding of diffuse alveolar damage (DAD) and occur in less than 20% of PM and DM patients with ILD [152] and are often accompanied by fever and malaise. The occurrence of rapidly progressive ILD has been well noted in patients with so-called amyopathic dermatomyositis (ADM) who have the typical rash of DM (Gottron's papules and/or heliotrope rash) but without muscle symptoms [157, 158]. ILD in these patients characteristically responds poorly to even aggressive treatment and shows high mortality rate [157]. Overall 5-year survival rates of patients with myositis-associated ILD are around 70% [6, 152].

Although NSIP is the most predominant histologic pattern followed by UIP [6, 63, 154], other histologic patterns have been frequently observed in patients with myositis-associated ILD, including COP and DAD [159]. Furthermore, ILD in myositis tends to exhibit a mixture of more than one histological pattern [63]. Treatment response of ILD varies depending on the underlying histological pattern [159]. COP responds favorably to steroids, whereas DAD and UIP respond poorly to immunosuppressive therapies with poor prognoses [152, 159]. The response of NSIP to steroids depends on degrees of inflammation and fibrosis [6, 63].

As in SSc, myositis specific autoantibodies tend to be mutually exclusive and are associated with distinctive clinical subsets. Other than anti-ARS antibodies, anti-melanoma differentiation-associated gene 5 (anti-MDA5) antibodies are associated with rapidly progressive ILD and/or ADM [160, 161]. Anti-ARS associated ILD shows more chronic course than anti-MDA5 associated ILD [161].

### 4.2. Genetics of Myositis and Myositis-Associated ILD

The two GWAS in myositis of European ancestry have shown that the strongest peak in Manhattan plot resides in MHC region of HLA 8.1 ancestral haplotype [162, 163]. Other suggestive genes outside of MHC region were PLCL1, BLK, and CCL21 in DM [163]. A GWAS imputation study also revealed TYK2 and FAM167A-BLK region as a susceptibility locus in DM [164]. FAM167A-BLK polymorphism was confirmed in Japanese myositis patients [165] and in Chinese patients with myositis or myositis-ILD [166]. A large scale immunochip study has shown that PTPN22, UBE2L3, CD28, TRAF6, and STAT4 are associated with myositis of Caucasian descents [167]: PTPN22 was primarily associated with PM. Other suggestive associations were IL18R1 and RGS1 in PM and GSDMB in DM. STAT4 had been shown to associate with myositis in a Japanese population [168].

Only a limited number of genes were examined against ILD phenotype. In large scale case-control studies in a Chinese Han population using candidate gene approach, CCL21, a myositis susceptibility gene in Caucasians [163], was found to associate with PM or PM-associated ILD [169], TNFAIP3 and IRF5 with myositis or myositis-associated ILD [170], and PLCL1 with DM and DM-associated ILD [171].

### 4.3. Treatment of Myositis-Associated ILD

High dose steroid therapy has been the primary treatment for myositis-associated ILD. The responsive rate against steroid monotherapy has been reported to be 50% [156]. Nowadays, various immunosuppressives are being used to treat myositis-associated ILD in combination with steroids, such as CYC, cyclosporine, or MMF. However, the drug effectiveness has been typically reported in retrospective observational studies of small sample size and/or case series without randomized controlled trials. Retrospective uncontrolled studies have reported effectiveness of rituximab in the setting of ILD [46, 172]. Tocilizumab, abatacept, or sifalimumab are under investigation for their efficacy in myositis [173].

## 5. Lung Involvement in Systemic Lupus Erythematosus (SLE)

The prevalence of lung involvement varies depending on the population and the detection method, as in other rheumatic diseases. Pulmonary function and HRCT abnormalities are common [174–176], but many are asymptomatic [174, 176]. As in RA, pulmonary manifestations related to SLE can affect any component of the lung structure from pleura, vasculature, parenchyma, and airways [174–176].

### 5.1. SLE Associated Pleural Disease

Pleural involvement constitutes one of the classification criteria of SLE. Clinically apparent pleural effusions have been observed in up to 50% of SLE patients with a prevalence of up to 93% at necropsy [177]. Effusions are either bilateral or unilateral. The pleural fluids in lupus pleuritis are often exudative with increased leukocyte cell count (polymorphonuclear neutrophils or lymphocytes predominant). The glucose level could be either normal or low as in lupus pleuritis [178]. Checking antinuclear antibodies (ANAs) of pleural fluid may be helpful for differential diagnosis of effusions, and a high titer of ANA (≥1/160) in pleural fluid is strongly indicative of SLE pleuritis [179]. SLE pleuritis responds favorably to NSAIDs for mild cases. For moderate to severe effusions, oral steroids are generally effective. Systemic immunosuppressive agents are infrequently indicated.

Shrinking lung syndrome is a rare manifestation of SLE, characterized by dyspnea, pleuritic chest pain, and a progressive decrease in lung volumes as reflected in restrictive PFT or diaphragmatic elevation, with no parenchymal or pulmonary vascular lesions. The reported prevalence of shrinking lung syndrome is around 1% [180, 181]. The pathogenesis of shrinking lung syndrome is unclear but several hypothetical models have been proposed without confirming evidence: microatelectasis associated with decreased surfactants, respiratory muscle weakness, diaphragm fibrosis and phrenic nerve palsy, and pleural inflammation. In particular, the most recent hypothesis claims that pleural inflammation or pleuritis has been proposed to cause diaphragmatic dysfunction with chronic hypoinflation and subsequent lung remodeling towards loss of compliance [182]. Although there are no standardized treatment regimens of shrinking lung syndrome, moderate-to-high dose steroids are used as first-line drugs with good success rates [181]. Immunosuppressives are combined if steroid treatment fails or from the beginning for severe cases. Successful use of rituximab in steroid-refractory cases has been consistently reported [181]. Theophylline and *β*-agonists have been tried to improve diaphragmatic strength, showing efficacy in 15% to 30% of patients. Overall, majority of patients show significant improvement in symptoms and PFT and long-term prognosis is favorable without resulting in respiratory failure or disease associated mortality [181].

### 5.2. SLE Associated Vascular Disease of the Lung

The vascular involvements of the lung in SLE patients include PAH and DAH. The prevalence of SLE associated PAH (SLE-PAH) has been typically around 4-5% [183–185]. Anti-cardiolipin antibodies (or lupus anticoagulant), Raynaud's phenomenon, or anit-U1 RNP antibodies were found to associate with PAH in SLE [184–186]. One-year survival of SLE patients with PAH from REVEAL registry was 94%, better than SSc-PAH (82%) and similar to RA-PAH (96%) [135]. Vasodilating treatment for SLE-PAH is similar to that for idiopathic PAH. However, additional immunosuppressive agents (steroids or CYC) seem to be beneficial in SLE-PAH [187–189].

DAH is a rare but devastating complication of SLE and its prevalence has been <2% of patients with SLE [190]. The onset of DAH is abrupt and the symptoms usually develop over hours to a few days. DAH could be either the presenting feature or observed in known SLE patients during a generalized lupus flare with associated multisystem involvement [190]. The mortality may approach beyond 50% [190, 191]. Majority of patients are treated with high dose of steroids in combination with immunosuppressives with or without plasma exchange and/or intravenous immunoglobulin. Several case series have shown the successful treatment of SLE-DAH with CYC [191] or rituximab [192].

### 5.3. SLE Associated Parenchymal Disease

ILD is less common in SLE than in other CTDs, with clinically significant cases observed in less than 8% of SLE population [193]. NSIP appears to be most common, while UIP pattern is uncommon [24]. When ILD is active with symptoms or progression, high dose steroids and immunosuppressive agents such as azathioprine, CYC, or MMF could be used.

### 5.4. SLE Associated Airway Disease

Airway disease is uncommon manifestation in SLE. However, upper and lower airways are both potential targets. Upper airway disease may range from mild ulceration, vocal cord paralysis, cricoarytenoid arthritis, and necrotizing vasculitis with airway obstruction [194]. Coexistence of SLE and BE on chest HRCT has been reported, though its clinical significance is uncertain [174–176]. SLE cases with bronchiolitis obliterans and COP have been rarely described [177].

## 6. Management Strategies on How to Screen and Monitor Lung Involvements in Rheumatic Diseases

**Figure 3:**
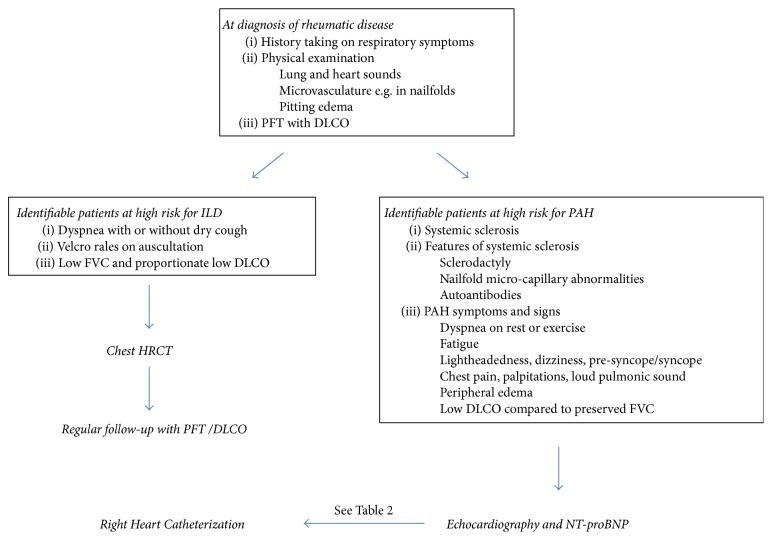
A schematic picture on screening and monitoring rheumatic disease associated lung involvements. DLCO: diffusing capacity of carbon monoxide; FVC: forced vital capacity; HRTC: high resolution computed tomography; ILD: interstitial lung disease; NT-proBNP: N-terminal probrain natriuretic peptide; PAH: pulmonary arterial hypertension; TR: tricuspid regurgitation.

Figure 3 shows a schematic picture on when or how to screen and monitor patients with rheumatic disease associated lung involvements focusing on ILD and PAH. Due to the impact of lung involvements on the patient prognosis, meticulous history taking on respiratory symptoms and relevant physical examinations are mandatory at the time of diagnosis of each rheumatic disease. Noninvasive PFTs are often recommended at diagnosis regardless of respiratory symptoms based on high frequency of subclinical involvements. However, because of the considerable heterogeneities in onset, involved structural components, severity, and prognosis of rheumatic disease associated lung diseases, there is no clear consensus on when and how to screen and monitor them. Although many physicians perform chest HRCT usually upon new onset or worsening of respiratory symptoms, auscultatory Velcro rales or abnormalities on the chest radiograph or PFTs appear, and the use of chest HRCT at the time of diagnosis has been stressed upon by some experts based on the high prevalence and prognostic implications of lung parenchymal involvements, particularly ILD, in rheumatic diseases. However, due to radiation exposure and nonprogressive nature in a significant proportion of asymptomatic patients, the use of HRCT as mandatory initial work-up and/or for periodic monitoring is controversial despite its better performance in detecting ILD than PFTs. Thus, its use has been justified for those at high risk identified by symptoms and signs (Figure 3). Monitoring is done using PFTs with or without chest radiography at a frequency depending on the severity and progression rate of lung diseases. Annual follow-up would be sufficient for asymptomatic mild cases while monthly or even shorter follow-up is required for rapidly progressive cases. In case of PAH, since symptoms are nonspecific and physical examination/imaging studies are not much helpful, early detection is difficult while early treatment would improve patient quality of life and prognosis. PAH should be screened using echocardiography, PFTs, and NT-proBNP in particular situations even asymptomatic, in case of SSc and SSc spectrum disorders (Table 2, Figure 3). Any rheumatic disease patients with PAH symptoms/signs should also be screened. Once patients show highly suggestive findings with the above modalities, PAH diagnosis should be made by RHC. Regarding airway diseases, their impact on the prognosis lacks data except for BE and there have been no recommendations of work-up or follow-up for this type of involvement.

## 7. Conclusion

Each rheumatic disease exhibits a unique pattern of lung involvements in terms of the affected lung structure and corresponding prevalence and incidence, severity, and treatment response. Although lung involvements, particularly ILD and PAH, are associated with significant morbidity and mortality in rheumatic diseases, advanced screening modalities enable us to detect and treat them early. However, considerably variable course of lung diseases from inactive state to fatal progression even among those within the same histologic group and underlying rheumatic disease makes it difficult to establish a uniform treatment strategy. To meet this need, it is of importance to understand the epidemiological characteristics associated with lung involvements of a given rheumatic disease and to identify high risk patients who bear clinical equipoise between potentially toxic treatments and disease progression. Furthermore, understanding the different genetics working in each lung involvement of a specific rheumatic disease will help us develop targeted therapies stemming from the underlying pathogenesis.

## Figures and Tables

**Table 1:** Spectrum and relative prevalence of lung involvements in rheumatic diseases.

	Parenchymal		Pleural	Vascular	
	ILD	Airways		PAH	DAH
Rheumatoid arthritis	++	++	++	+	–
Systemic sclerosis	+++	–	–	+++	–
Myositis	+++	–	–	+	–
Systemic lupus erythematosus	+	+	+++	+	++

The signs show relative prevalence of each manifestation (none: –, low: +, medium: ++, and high: +++); ILD: interstitial lung disease; DAH: diffuse alveolar hemorrhage; PAH: pulmonary arterial hypertension (cited and modified from “Interstitial Lung Disease in Connective Tissue Disorders” by A. Fischer and R. du Bois. Lancet 2012; 380: 689–98).

**Table 2:** Recommendations for right heart catheterization for SSc and SSc-spectrum disorder.

Modalities	Parameter thresholds required for RHC	Signs/symptoms^*∗*^ required for RHC
Echocardiography	TR velocity	
	2.5–2.8 m/s	Yes
	>2.8 m/s	No
	Cavity enlargements irrespective of TR velocity	No
	Right atrial major dimension > 53 mm or	
	Right ventricular mid-cavity dimension > 35 mm	

Pulmonary function tests	FVC/DLCO ratio > 1.6 and/or DLCO < 60%^*∗∗*^	Yes
	FVC/DLCO ratio > 1.6 and/or DLCO < 60% and NT-pro BNP > 2 times upper limit of normal^*∗∗*^	No

Composite measures	Meets DETECT algorithm in patients with DLCO < 60% and disease duration of >3 years from 1st non-Raynaud's symptom	No

^*∗*^Symptoms: dyspnea on rest or exercise, fatigue, presyncope/syncope, chest pain, palpitations, dizziness, and lightheadedness. Signs: loud pulmonic sound and peripheral edema. ^*∗∗*^Without overt systolic dysfunction, greater than grade I diastolic dysfunction or greater than mild mitral or aortic valve disease, or evidence of PAH in echocardiography; DLCO: diffusing capacity of carbon monoxide; FVC: forced vital capacity; NT-proBNP: N-terminal probrain natriuretic peptide; PAH: pulmonary arterial hypertension; SSc: systemic sclerosis; TR: tricuspid regurgitation (cited and modified from “Recommendations for Screening and Detection of Connective Tissue Disease-Associated Pulmonary Arterial Hypertension” by D. Khanna, C. H. Tseng, N. Farmani et al. Arthritis Rheum 2013; 65: 3194-201).
